# Interleukin-9 promotes early mast cell-mediated expulsion of *Strongyloides ratti* but is dispensable for generation of protective memory

**DOI:** 10.1038/s41598-018-26907-2

**Published:** 2018-06-05

**Authors:** Martina Reitz, Wiebke Hartmann, Nikolas Rüdiger, Zane Orinska, Marie-Luise Brunn, Minka Breloer

**Affiliations:** 1 Bernhard Nocht Institute for Tropical Medicine, Hamburg, Germany; 2 Division of Experimental Pneumology, Research Center Borstel, Borstel, Germany

## Abstract

IL-9 is a cytokine with pleiotropic function that mediates allergic inflammation and immunity to intestinal helminth parasites. Accumulating evidence suggests that IL-9 acts via both, initiation and regulation of adaptive immune responses and direct activation of intestinal effector pathways. Here we use IL-9 receptor deficient mice on BALB/c and C57BL/6 genetic background to dissect effector and regulatory functions of IL-9 during infection with the parasitic nematode *Strongyloides ratti*. IL-9 receptor-deficient mice displayed increased intestinal parasite burden and prolonged infection irrespective of the genetic background of the mice. Increased parasite burden was correlated to a reciprocally reduced early degranulation of mucosal mast cells, reduced intestinal IL-13 expression and caused by IL-9 receptor deficiency on hematopoietic cells. We observed additional significant changes in the adaptive immune response to *S. ratti* infection in the absence of the IL-9 receptor that depended on the mouse strain. However, the generation of protective memory to a second infection was intact in IL-9 receptor-deficient mice, irrespective of the genetic background. In summary, our results support a central role for IL-9 as an early mast cell activating effector cytokine during intestinal helminth infection while non-redundant functions in the initiation and amplification of adaptive immune responses were not apparent.

## Introduction

Soil-transmitted intestinal helminth parasites infect approximately one quarter of the world’s population^1^. Among the dominant species *Ascaris lumbricoides*, *Trichuris trichuria*, *Ancylostoma duodenale* and *Necator americanus*, the roundworm *Strongyloides stercoralis* is responsible for 30 to 100 million infected people worldwide^2,3^.

We use the rodent-specific *Strongyloides ratti* as a model for an intestinal parasite with tissue migrating life stages^4^. Free-living infective third stage larvae (L3i) actively penetrate the skin of their mammalian host. Subsequently, L3i migrate within 2 days on yet not fully defined routes via the skin, muscle and partially via the lung to the head and mouth^5^. After being swallowed, L3i reach the small intestine by day 3 post infection (p.i.) and moult via L4 to parasitic adults by day 5–6 p.i. Adults live embedded in the intestinal mucosa and reproduce by parthenogenesis. Eggs as well as hatched first stage larvae (L1) leave the host with the faeces to complete the life cycle.

Immune competent mice terminate *Strongyloides* infection within one month in the context of a canonical type 2 immune response and remain semi-resistant to subsequent infections^4^. Tissue migrating larvae are opsonized by complement and antibodies (Ab) and killed by eosinophil and neutrophil granulocytes^6^, while final expulsion of adults from the intestine is predominantly mediated by mucosal mast cells^7,8^. In addition to the dominant T helper 2 (Th2) cell-derived effector cytokines IL-4 and IL-13^9,10^, accumulating evidence demonstrated a central role for IL-9 in mediating immunity to intestinal helminth parasites in the mouse system. Expulsion of *Trichuris muris*^11–13^, *Trichinella spiralis*^14–16^ and *S. ratti*^17,18^ was promoted by IL-9.

Interestingly, the impact of IL-9 on the expulsion of *Nippostrongylus brasiliensis* depended on the genetic background of the infected mice. While IL-9 deficient BALB/c mice displayed increased intestinal *N. brasiliensis* burden^19^, IL-9 deficient 129 × C57BL/6 (F_2_) mice had unchanged intestinal parasite burden despite impaired mastocytosis during infection^19,20^. The genetic background of the host was also relevant for the regulation of IL-9 production during intestinal helminth infection. We have shown that specifically IL-9 production was targeted by *S. ratti*-induced immune evasive strategies that differed in BALB/c and C57BL/6 mice^17,18^. *S. ratti* infection led to an expansion of Foxp3^+^ regulatory T cells (Treg) in both mouse strains. However, depletion of Treg resulted in elevated IL-9 production and reduced parasite burden selectively in BALB/c mice, whereas C57BL/6 mice displayed unchanged IL-9 production and parasite burden in the absence of Treg^17,21^. *S. ratti-*infected C57BL/6 mice displayed increased expression of the co-inhibitory receptor B and T Lymphocyte Attenuator (BTLA) on CD4^+^ T cells in addition to their expanded Treg numbers^18^. The deletion of either BTLA or its ligand finally elevated IL-9 production and reduced parasite burden in *S. ratti-*infected C57BL/6 mice, thus suggesting that additional regulatory pathways suppressed IL-9 production during helminth infection on this genetic background.

Although the multiple pathways triggered via IL-9 are still being investigated, several lines of evidence show that IL-9 functions as both: an intestinal effector cytokine and an initiator and regulator of type 2 and type 17 immune responses^22–24^. Specifically the impact of IL-9 in the generation of protective type 2 memory responses to parasite infection has not been investigated so far.

Here we use Interleukin-9 receptor (IL-9R)-deficient BALB/c and C57BL/6 mice to dissect the putative roles of IL-9 in mice of different genetic background and during the initiation and the execution of anti-helminth immune responses. We show that IL-9 functioned as a non redundant mast cell activating effector cytokine, induced intestinal IL-13 transcription and promoted parasite expulsion from the intestine irrespective of the genetic background of the host. Although the absence of IL-9R-mediated signalling modulated T and B cell responses during infection to some extent, functional IL-9R was not essential for the establishment of protective immune memory against a second *S. ratti* infection.

## Results

### IL-9R signalling on hematopoietic cells is central for the mast cell mediated early eradication of *S. ratti* infection in BALB/c and C57BL/6 mice

**Figure 1:**
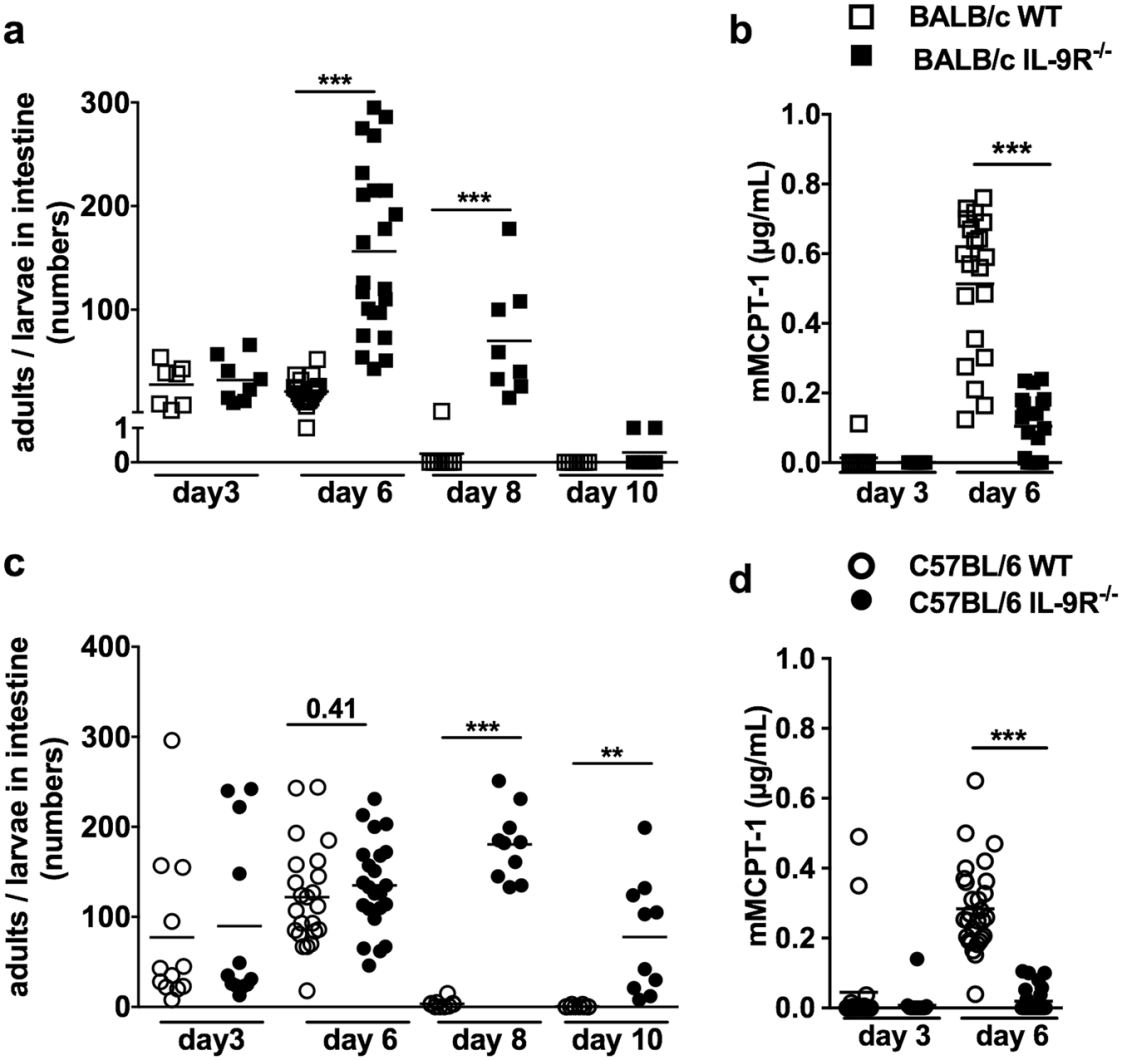
Increased *S. ratti* parasite burden and reduced mast cell degranulation in IL-9R^−/−^ BALB/c and C57BL/6 mice. WT (open symbols) and IL-9R^−/−^ (closed symbols) mice on BALB/c (**a**,**b**, squares) or C57BL/6 (**c**,**d**, circles) background were s.c. infected with 2000 *S. ratti* L3i. (**a,c**) Parasites in the small intestine were counted at indicted time points. (**b**,**d**) mMCPT-1 in the serum was quantified at indicated time points. Shown are combined results from 2–4 independent experiments n ≥ 4 per group, time point, and experiment. Each symbol represents an individual mouse, the line shows the mean and asterisks indicate statistical significant differences of the mean between WT and IL-9R^−/−^ mice (^**^p < 0.01; ^***^p < 0.001 Students *t*-test).

To analyse the impact of IL-9 during control of *S. ratti* infection, we compared intestinal parasite burden in wildtype (WT) and IL-9R^−/−^ mice of BALB/c and C57BL/6 genetic background (Fig. 1). *S. ratti* L3 migrate within 3 days after s.c. injection into the footpad of experimental mice via the tissue and head region to the small intestine^5^. Parasites subsequently embed themselves into the mucosa and moult to parasitic adults by day 5–6 p.i. While numbers of “arriving” *S. ratti* L3 in the intestine were alike day 3 p.i., IL-9R deficiency strongly increased adult *S. ratti* parasite burden at later time points in both mouse strains, although with different kinetics. Elevated parasite burden in IL-9R^−/−^ BALB/c mice were recorded at days 6 and 8 p.i. (Fig. 1a) whereas IL-9R^−/−^ and WT C57BL/6 mice had comparable parasite numbers at day 6 p.i. and displayed a significant elevation in intestinal parasite burden at later time points i.e. days 8 and 10 p.i. (Fig. 1c).

As efficient expulsion of *S. ratti* from the intestine is mediated by mucosal mast cells^7^ and mast cells are dominant targets of IL-9^20^, we quantified mast cell activation. To this end we measured mouse mast cell protease 1 (mMCPT-1) that is released specifically by mucosal mast cells upon degranulation^25^, in the sera of infected mice. Both IL-9R^−/−^ BALB/c (Fig. 1b) and IL-9R^−/−^ C57BL/6 mice (Fig. 1d) showed drastically reduced concentrations of mMCPT-1 in the serum, compared to WT mice.

**Figure 2:**
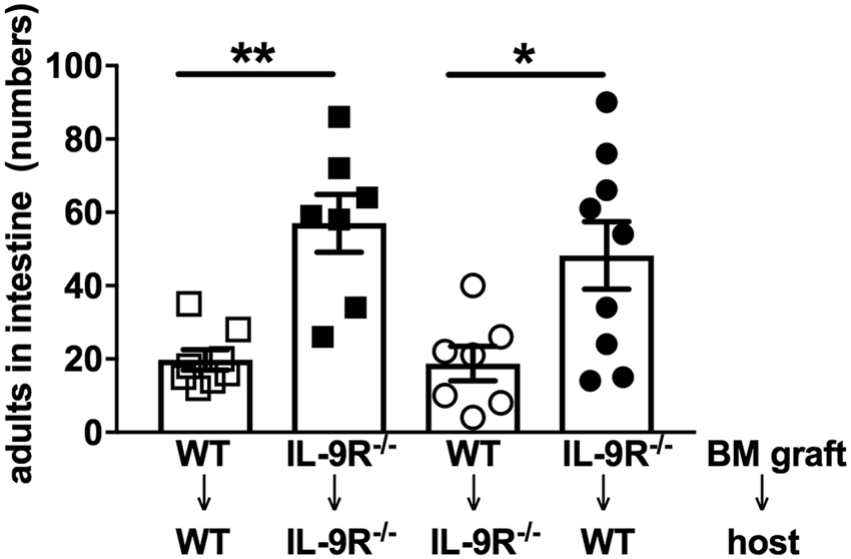
Increased *S. ratti* parasite burden in mice with IL-9R^−/−^ hematopoietic compartment. WT and IL-9R^−/−^ mice on BALB/c background were lethally irradiated with 8 Gy and received 3 × 10^6^ bone marrow cells from WT or IL-9R^−/−^ donor mice as indicated. Engraftment was verified by flow cytometry after 8 weeks and chimeras were s.c. infected with 2000 *S. ratti* L3i. Parasite burden in the small intestine was counted at day 6 p.i. Shown are the combined results of 2 independent experiments n ≥ 3 per group and experiment. Each symbol represents an individual mouse, error bars show SEM and asterisks indicate statistical significant differences between the groups (^*^p < 0.05; ^**^p < 0.01; Students *t*-test).

The IL-9R is predominantly expressed on hematopoietic cells such as T cells, mast cells and group 2 innate lymphoid cells (ILC2)^22^, but also somatic epithelial cells were reported to respond to IL-9^26–28^. To distinguish between the consequences of IL-9R-mediated signalling on somatic and hematopoietic cells, we created bone marrow chimeras and performed *S. ratti* infection experiments (Fig. 2). Transplantation of IL-9R^−/−^ bone marrow into either IL-9R^−/−^ hosts or into WT hosts elevated intestinal parasite burden at day 6 of *S. ratti* infection. By contrast, IL-9R deficiency on somatic tissues did not change parasite burden in IL-9R^−/−^ chimeras transplanted with IL-9R competent WT bone marrow. This result shows that early expulsion of *S. ratti* from the intestine depended on IL-9R-mediated signalling selectively on hematopoietic cells.

**Figure 3:**
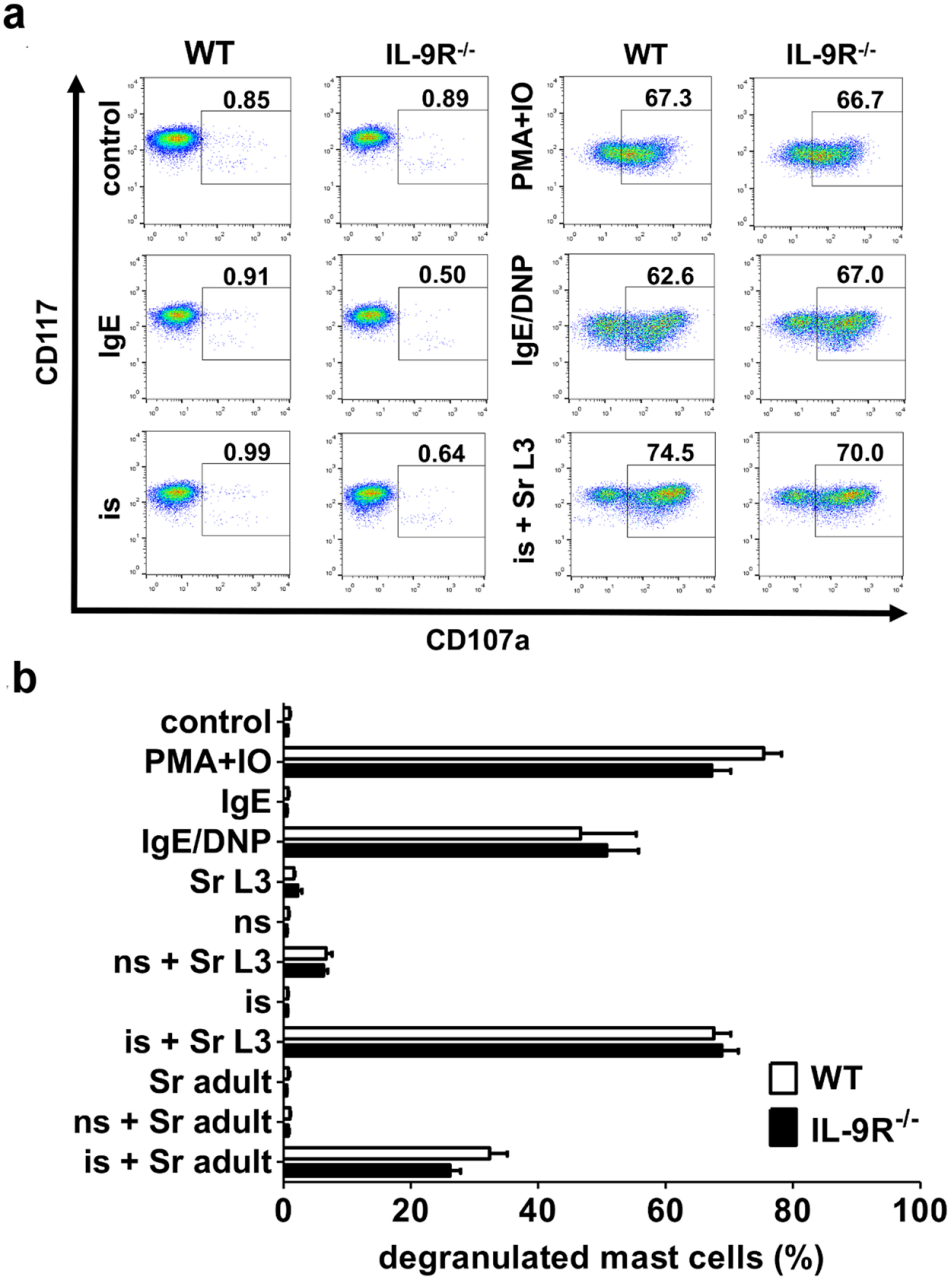
Intact degranulation of IL-9R^−/−^ BMMC in response to Ab-mediated stimulation. BALB/c WT and IL-9R^−/−^ BMMC were either sensitized with 0.2 µg/mL DNP-specific IgE (clone SPE-7) or 1:100 dilutions of naïve sera (ns) or *S. ratti-*specific immune sera (is; taken from day 21 re-infected BALB/c mice) or left untreated overnight. BMMC were washed in PBS and 2 × 10^5^ cells were either incubated with the crosslinking model antigen DNP-HSA (IgE/DNP, 20 ng/mL), or 100 µg/mL *S. ratti* L3 lysate (*Sr* L3), or 15 µg/mL *S. ratti* adult lysate (*Sr* adult) in round bottom 96-well-plates for 20 minutes at 37 °C. Negative control BMMCs were left unstimulated (control) and positive control BMMC were activated with 100 ng/mL PMA/Ionomycin (PMA + IO). Cells were stained for FcεRIα, CD107a and CD117 expression and analyzed on a BD Calibur Flow cytometer. (**a)** Representative dot plots showing CD117/CD107a expression on BMMCs. Numbers show frequency of degranulated BMMC identified as CD107a^+^ cells in response to indicated stimuli. (**b**) Graphs show combined results out of 2 independent experiments (n = 9–10 per group) presented as mean and error bars indicate SEM.

IL-9 is an important maturation and activation factor for mast cells^20,29^. To address the possibility that constitutive IL-9R deficiency would lead to infection-independent intrinsic defects in the mast cell compartment, we compared the degranulation response, quantified as CD107a translocation from secretory granules to the plasma membrane, of WT and IL-9R^−/−^ bone marrow-derived mast cells (BMMC) to different stimuli *in vitro* (Fig. 3). The percentage of degranulated mast cells in response to either non-specific, maximal stimulation via PMA/Ionomycin or FcεRI-mediated activation via DNP-specific IgE, cross-linked by polyvalent DNP, was comparable in WT and IL-9R^−/−^ BMMC. Also activation of BMMC by incubation with *S. ratti-*specific antiserum and antigen extract derived from *S. ratti* L3 or adult parasites, caused efficient and specific degranulation to the same extent in WT and IL-9R^−/−^ BMMC (Fig. 3b). These results show that the reduced degranulation of mast cells that we recorded during *S. ratti* infection of IL-9R^−/−^ mice *in vivo* (Fig. 1b,d) was due to impaired activation of the mast cells in the context of infection and not due to a generalized defect in their function.

### IL-9R deficiency results in deregulation of the immune response during *S. ratti* infection

**Figure 4:**
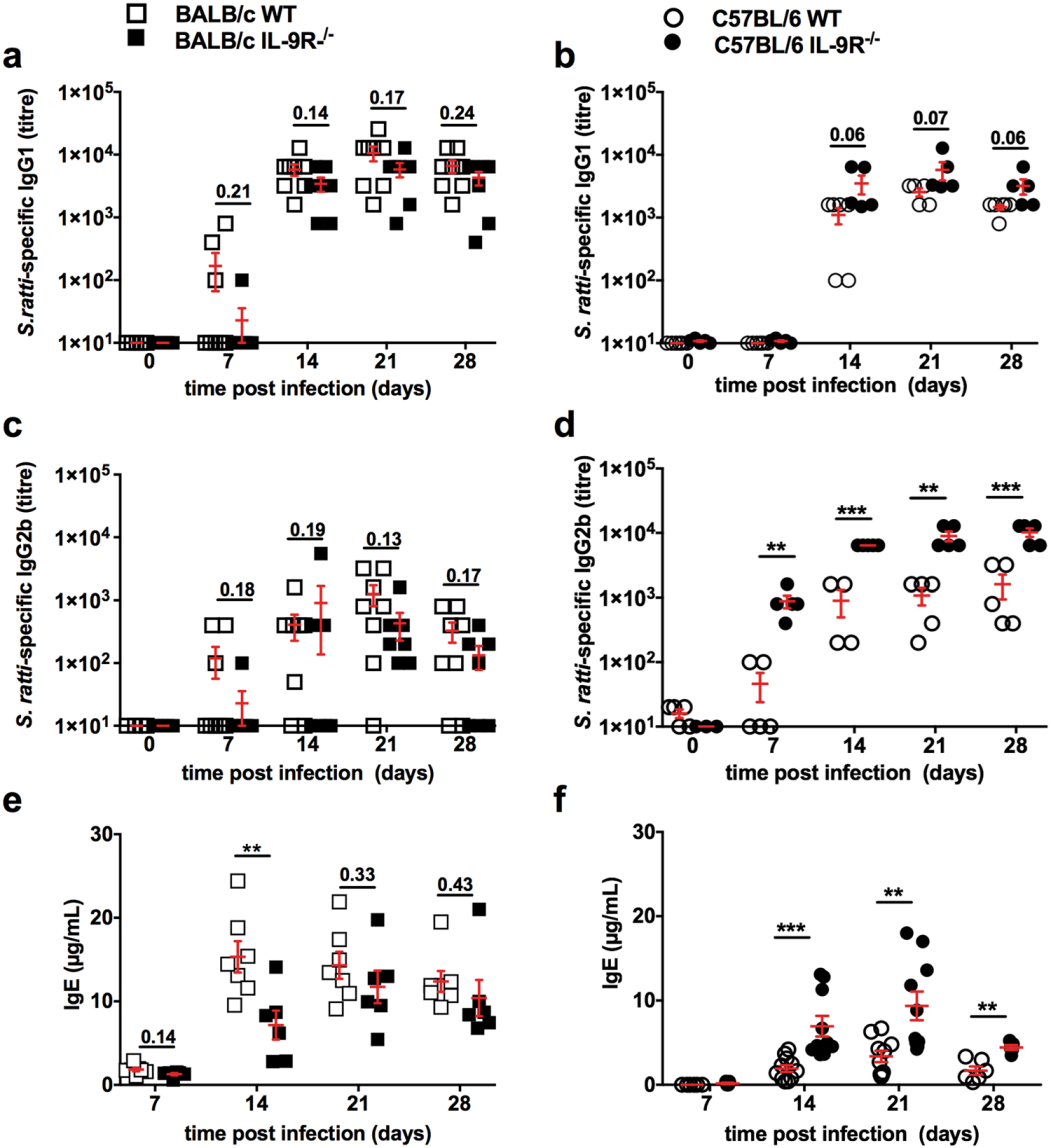
Deregulated humoral immune response to *S. ratti* infection in IL-9R^−/−^ mice. WT (open symbols) and IL-9R^−/−^ (closed symbols) mice on BALB/c (squares) or C57BL/6 (circles) background were s.c. infected with 2000 *S. ratti* L3i. Serum samples were obtained at the indicated time points and *S. ratti* antigen-specific IgG1 (**a**,**b**), IgG2b (**c**,**d**) and total IgE (**e**,**f**) in the serum was quantified by ELISA. Shown are combined results from 2 independent experiments n ≥ 4 per group, time point, and experiment. Each symbol represents an individual mouse the line shows the mean, error bars indicate SEM and asterisks indicate statistical significant differences of the mean between WT and IL-9R^−/−^ mice (^*^p < 0.05; ^**^p < 0.01; ^***^p < 0.001, Students *t*-test).

**Figure 5:**
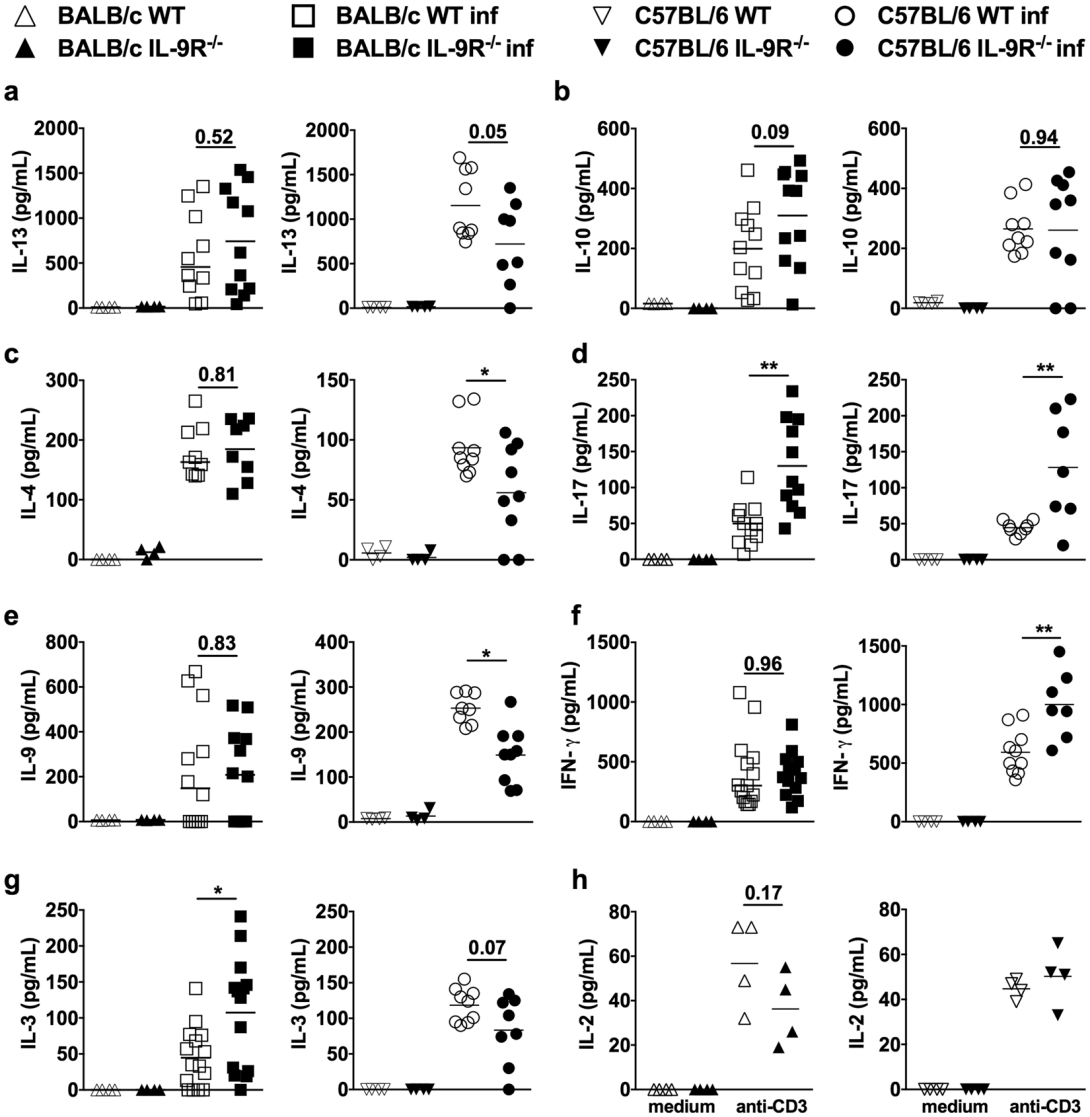
Deregulated cellular immune response to *S. ratti* infection in IL-9R^−/−^ mice. WT (open symbols) and IL-9R^−/−^ (closed symbols) mice on BALB/c (squares) or C57BL/6 (circles) background were s.c. infected with 2000 *S. ratti* L3i. Mice were sacrificed at day 6 p.i. and MLN cells were prepared. 1 × 10^6^ MLN cells derived from infected or naïve (rectangles) mice were stimulated in 96-well plates in triplicates in the presence of (**a**–**e**, **g**) *S. ratti* antigen lysate (20 µg/mL) or (**f**,**h**) anti-CD3 (1 µg/mL) for 72 h at 37 °C. Concentration of IL-13 (**a**), IL-10 (**b**), IL-4 (**c**), IL-17 (**d**), IL-9 (**e**), IFN-γ (**f**) and IL-2 (**h**) in the culture supernatants were quantified by ELISA. Shown are combined results from 2 (day 6 *S. ratti-*infected) or 1 (naïve mice) independent experiments n ≥ 4 per group, time point, and experiment. Each symbol represents an individual mouse, the lines show the mean and asterisks indicate statistical significant differences of the mean between WT and IL-9R^−/−^ mice (^*^p < 0.05; ^**^p < 0.01, Students *t*-test).

**Figure 6:**
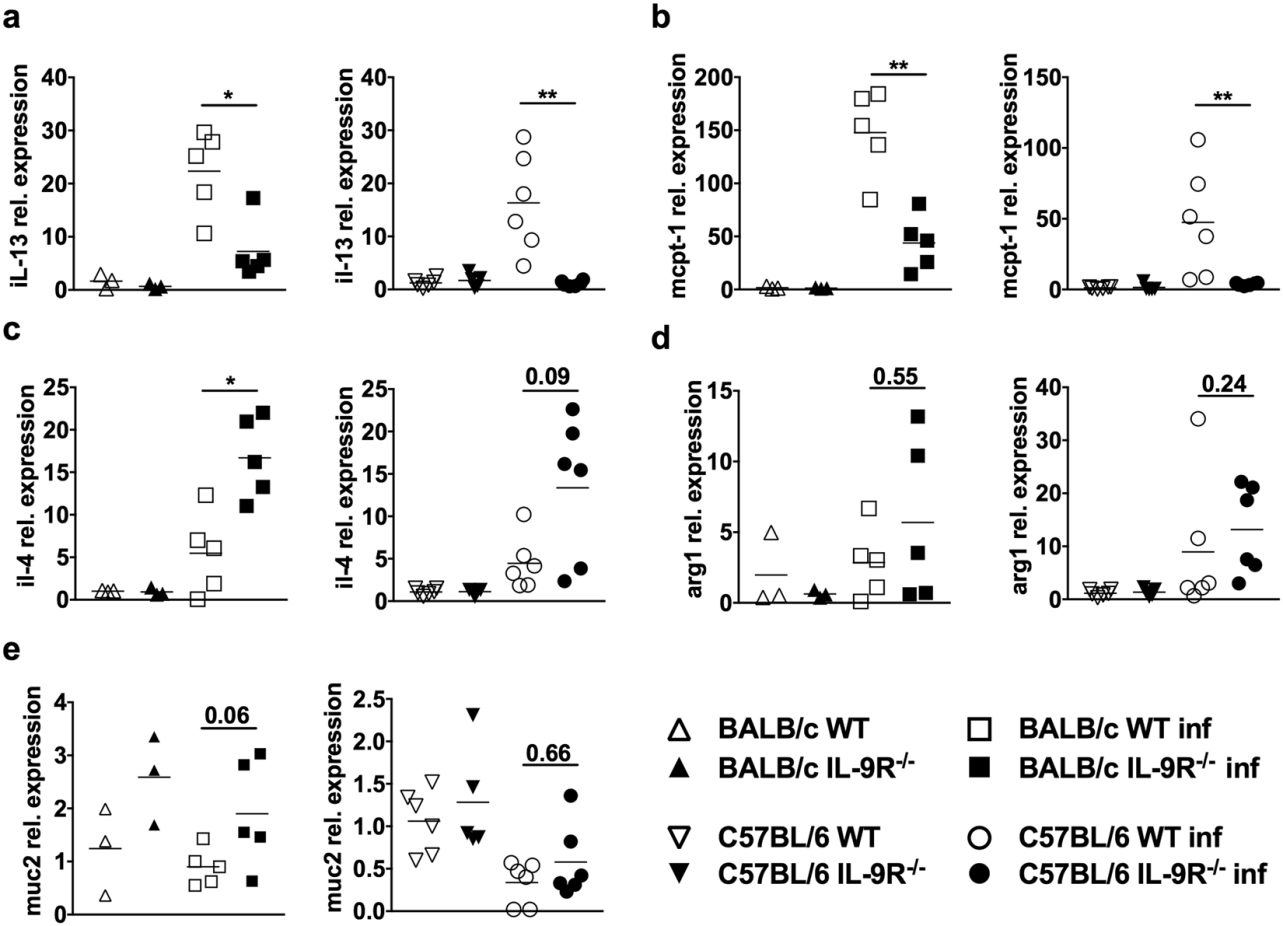
Intestinal mRNA expression in IL-9R^−/−^ mice during *S. ratti* infection. WT (open symbols) and IL-9R^−/−^ (closed symbols) mice on BALB/c (squares) or C57BL/6 (circles) background were s.c. infected with 2000 *S. ratti* L3i. Mice were sacrificed at day 6 p.i. and intestines were prepared. RNA was isolated from 1 cm samples of infected or naïve (rectangles) mice in duplicates. Relative expression of (**a**) IL-13, (**b**) mMCPT-1, (**c**) IL-4, (**d**) Arg1 and (**e**) muc2, mRNA in the small intestine was quantified by qPCR in duplicates and calculated using the comparative Ct method. Results are expressed as 2^−ΔΔCt^ using GAPDH as housekeeping gene and naïve mice as reference group. Graphs show combined results out of 2 independent experiments with n ≥ 3 per group, time point, and experiment. Each symbol represents an individual mouse, the lines show the mean and asterisks indicate statistical significant differences of the mean between WT and IL-9R^−/−^ mice (^**^p < 0.01; ^***^p < 0.001, Students *t*-test). For naïve BALB/c mice 1 experiment with n = 3 was performed, for day 6 *S. ratti-*infected BALB/c mice 2 independent experiments with n = 3 and n = 2 were performed.

The reduced mast cell activation observed in *S. ratti*-infected IL-9R^−/−^ mice could either reflect a direct function of IL-9 as a mast cell co-activating effector cytokine and/or an indirect function of IL-9 in initiating and shaping the adaptive T and B cell immune response^22,23^. To address this question we compared the quality and quantity of the humoral (Fig. 4), cellular (Fig. 5) and intestinal (Fig. 6) immune response to *S. ratti* infection in WT and IL-9R^−/−^ mice on the BALB/c and C57BL/6 background.

The antigen-specific IgG1 response that is dominant during *S. ratti* infection^30^ was not modulated in the absence of IL-9R on BALB/c background (Fig. 4a) and elevated by trend in IL-9R^−/−^ C57BL/6 mice (Fig. 4b). Antigen-specific IgG2a was not detectable (data not shown) and IgG2b was unchanged in IL-9R^−/−^ BALB/c (Fig. 4c) but elevated in IL-9R^−/−^ C57BL/6 mice (Fig. 4d) compared to WT mice. Quantification of antigen-specific IgE in IgG1-depleted serum samples was not possible due to limited serum samples but quantification of polyclonal IgE, that increased during *S. ratti* infection, showed a reduction in IL-9R^−/−^ BALB/c mice (Fig. 4e) and a reciprocal increase in IL-9R^−/−^ C57BL/6 mice (Fig. 4f) compared to WT mice.

To study the cellular immune response, mesenteric lymph node (MLN) cells derived from either naïve or day 6 *S. ratti*-infected mice were re-stimulated *ex vivo* with *S. ratti* antigen and subsequent cytokine production was compared in WT and IL-9R^−/−^ mice (Fig. 5). Selectively MLN cells derived from *S. ratti*-infected mice and not from naïve mice produced cytokines in response to *S. ratti* antigen, as expected. Therefore, as an additional control for viability, naïve MLN cells were stimulated with anti-CD3 to induce IL-2 secretion (Fig. 5h). Th2 associated IL-13 and IL-4 release was not modulated by IL-9R deficiency in infected BALB/c mice but reduced in IL-9R^−/−^ C57BL/6 mice (Fig. 5a,c). Production of IL-9 and of IL-3, another important activator of mast cells and involved in the control of *Strongyloides* infection^31^, were unchanged or elevated in IL-9R^−/−^ BALB/c and either significantly (IL-9) or by trend (IL-3) reduced in IL-9R^−/−^ C57BL/6 mice (Fig. 5e,g). Both, IL-9R^−/−^ BALB/c and IL-9R^−/−^ C57BL/6 mice displayed unchanged production in antigen-specific IL-10 (Fig. 5b) and increased antigen-specific IL-17 production during *S. ratti* infection (Fig. 5d). Since no significant amounts of *S. ratti* antigen-specific IFN-γ were produced (data not shown), we compared the IFN-γ release in response to anti-CD3 stimulation of MLN cells. Specifically MLN cells derived from *S. ratti*-infected mice produced IFN-γ that was not altered by IL-9R deficiency in BALB/c mice and elevated in IL-9R^−/−^ C57BL/6 mice compared to WT mice (Fig. 5f). These combined findings show that IL-9R deficiency during *S. ratti* infection of BALB/c mice impaired humoral type 2 responses (IgE) while the cellular type 2 response in MLN cells (IL-13, IL-4) remained unchanged, despite the increased parasite burden in these mice at the time point of analysis. IL-9R-deficient C57BL/6 mice displayed elevated humoral responses of all isotypes i.e. type 1 associated IgG2b as well as type 2 associated IgE. By contrast, type 2 associated cytokine production in MLN cells derived from IL-9R-deficient C57BL/6 mice was reduced at day 6 p.i., a time point where parasite burden was still comparable in WT and IL-9R^−/−^ C57BL/6 mice.

To analyse the local immune response, we quantified mRNA expression in small intestines derived from naïve and day 6 *S. ratti-*infected WT and IL-9R-deficient mice (Fig. 6). *S. ratti* infection induced the transcription of IL-13 mRNA in WT mice that was drastically reduced by IL-9R-deficiency (Fig. 6a). Interestingly, up-regulation of IL-4 and Arginase 1 mRNA were not abrogated by IL-9R deficiency but even increased in IL-9R-deficient BALB/c and C57BL/6 mice, suggesting that type 2 immunity could be established in principle in the absence of IL-9R (Fig. 6c,d). Transcription of mucins such as mucin 2 was neither affected by IL-9R deficiency nor induced during *S. ratti* infection (Fig. 6e). In line with the observed reduction of systemic mMCPT-1 protein concentration in the serum of *S. ratti-*infected IL-9R^−/−^ BALB/c and IL-9R^−/−^ C57BL/6 mice (Fig. 1b,d), also transcription of mMCPT-1 in the small intestine was reduced (Fig. 6b). As mucosal ILC2 represent a major IL-9 responsive source of IL-13 and ILC2 have been involved in immunity to *S. venezuelensis*^32^, we quantified ILC in the Lamina Propria and Peyer’s Patches of WT and IL-9R^−/−^ BALB/c mice. Surprisingly, IL-9R-deficient mice and WT mice displayed comparable frequencies of group 1, 2, and 3 ILC day 4 p.i. (Supplementary Fig. S1), and responded to PMA/Ionomycin stimulation with comparable production of IL-13 an IL-5 (Supplementary Fig. S2). Thereby, day 4 p.i. was used as time point of analysis because preparation of viable cells from the intestine of day 6 *S. ratti* infected mice was not possible.

### BALB/c and C57BL/6 mice mount protective immune memory responses to *S. ratti* infection in the absence of IL-9R

**Figure 7:**
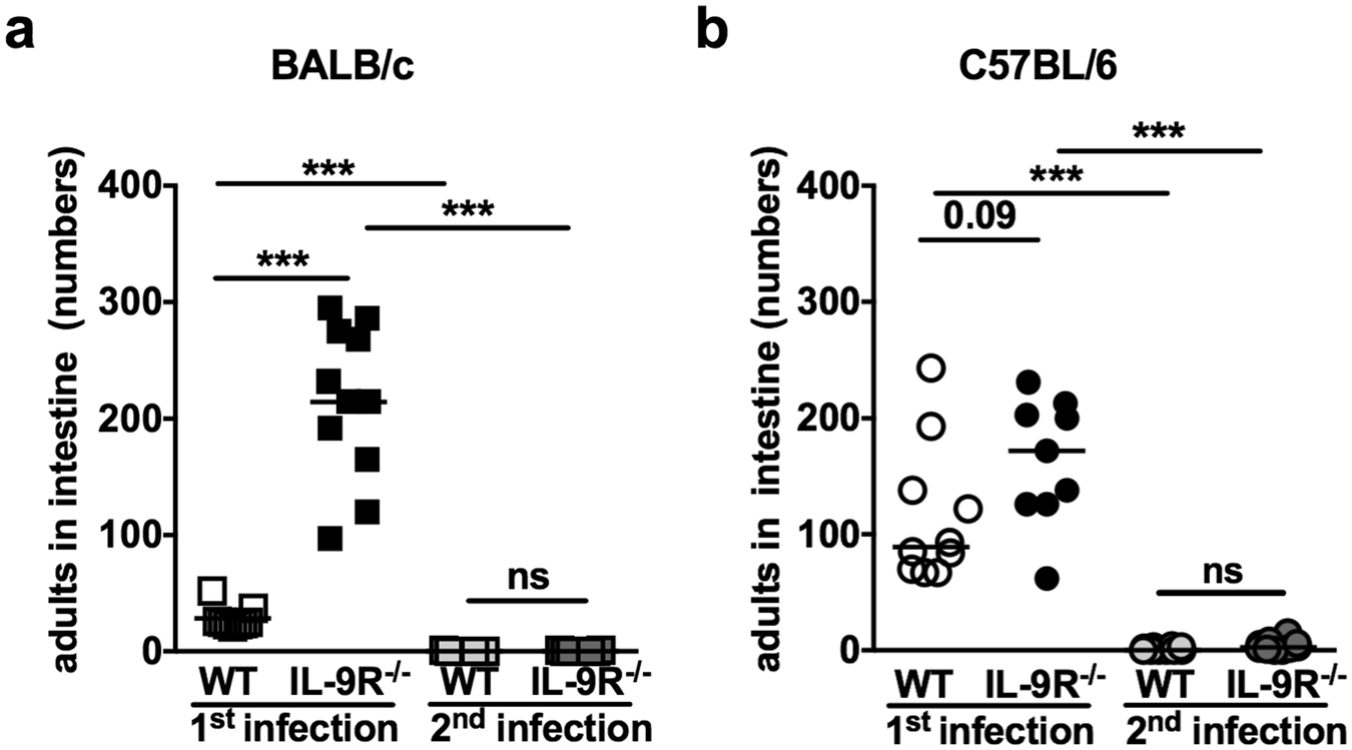
Generation of protective immunity to second *S. ratti* infection in IL-9R^−/−^ BALB/c and C57BL/6 mice. WT (white and light grey symbols) and IL-9R^−/−^ mice (black and dark grey symbols) on BALB/c (**a**, squares) or C57BL/6 (**b**, circles) background were s.c. infected with 2000 *S. ratti* irradiated L3i or left naïve. 4 weeks later the naïve (1^st^ infection) and the formerly infected (2^nd^ infection) mice were challenged by infection with 2000 vital L3i. Parasites in the small intestine were counted at day 6 p.i. Shown are combined results from 2 independent experiments n ≥ 4 per group, time point, and experiment. Each symbol represents an individual mouse, lines show the mean and asterisks indicate statistical significant differences of the mean between WT and IL-9R^−/−^ mice (^*^p < 0.05 ^**^p < 0.01; ^***^p < 0.001, Students *t*-test).

To test the quality of the adaptive immune response that was initiated in the absence of IL-9R in a more stringent approach, we measured the immune-mediated protection against a second infection. To this end WT and IL-9R^−/−^ mice were infected with irradiated *S. ratti* L3i and re-infected 28 days later together with a naïve control group receiving the first infection (Fig. 7). Numbers of adults in the intestine were significantly reduced in mice receiving a second infection compared to naïve mice receiving the first infection. Thereby, WT and IL-9R^−/−^ mice on BALB/c (Fig. 7a) and C57BL/6 (Fig. 7b) background were protected to the same extent. This result clearly shows that efficient and protective immunity to *S. ratti* infection was initiated and maintained in the absence of IL-9R despite the deregulated cytokine and antibody response observed in IL-9R^−/−^ mice during the first infection.

## Discussion

IL-9 is a cytokine with pleiotropic function that is predominantly produced by ILC2^33^ and Th9 cells^34^ and that contributes to the protective immunity against intestinal helminth parasites^24^. Here we show that IL-9R-mediated signalling is central for the early activation of mucosal mast cells, the early induction of intestinal IL-13 and the efficient expulsion of the helminth *S*. *ratti* from the intestine but that IL-9 is dispensable for the establishment of protective immune memory.

IL-9R-deficient mice on both, BALB/c and C57BL/6 genetic background displayed (i) increased and prolonged parasite burden in the small intestine; (ii) reduced early degranulation of mucosal mast cells; (iii) reduced intestinal IL-13 expression and (iv) complete protection against a second infection.

As mucosal mast cells are major targets of IL-9^20^ and mediate intestinal expulsion of *S. ratti*^7,8^, our combined results suggest that IL-9 represents a local effector cytokine that, directly or indirectly, promotes activation of mucosal mast cells in the wildtype situation, thereby contributing to efficient parasite expulsion.

In support of this hypothesis, we demonstrate that impaired host defence was due to IL-9R deficiency on hematopoietic cells. Bone marrow chimeras displaying WT somatic tissue and IL-9R-deficient hematopoietic cells had the same elevated *S. ratti* parasite burden in the intestine as IL-9R^−/−^ mice. Chimeras displaying IL-9R-deficient somatic tissue and WT bone hematopoietic cells phenocopied WT mice. Thereby, we rule out direct effects of IL-9 on intestinal epithelial cells and smooth muscle cells during *S. ratti* infection. An indirect activation of somatic cells via IL-9-dependent induction of IL-13 in hematopoietic cells, such as mast cells or ILC2, as shown for IL-9-induced asthma^35^, is still possible.

Indeed, we observed reduced intestinal expression of IL-13 in IL-9R-deficient BALB/c and C57BL/6 mice. IL-13 is a central intestinal effector cytokine that promotes intestinal defence pathways such as goblet cell derived mucus production, proliferation of intestinal cells and increased intestinal peristaltic i.e. the “weep and sweep reaction”^36^. However, the contribution of this reduced IL-13 expression to the phenotype of IL-9R-deficient mice may be limited. We have shown previously that day 6 *S. ratti-*infected mast cell-deficient and mast cell-competent mice displayed comparable IL-13 expression in the intestine despite drastically elevated parasite burden in the absence of mast cells^7^. Moreover, neutralisation of endogenous IL-13 did not change intestinal parasite burden whereas neutralisation of IL-9 increased parasite burden^17^, suggesting that IL-13-mediated effects are not as important for intestinal immunity to *S. ratti* as IL-9-mediated effects. Consenting with these findings, downstream mediators of anti-helminth immunity that are dependent on IL-13 such as mucins were not modulated by IL-9R deficiency. By contrast, intestinal mMCPT-1 mRNA, and systemic concentration of mMCPT-1, indicating mast cell activation and degranulation, was reduced in IL-9R-deficient mice.

We provide evidence that this delayed activation of IL-9R^−/−^ mast cells did not reflect intrinsic functional defects independent of the infection context because WT and IL-9R^−/−^ mast cells responded equally well to FcR-mediated stimulation with model antigens or *S. ratti-*derived antigens *in vitro*.

The delayed activation of mast cells during *S. ratti* infection and subsequent impaired host defence in IL-9R deficient mice could reflect either missing direct engagement of IL-9R on mast cells or an impaired initiation of the classical type 2 immune response. IL-9 functions as a central autocrine survival factor for ILC2 that initiate and amplify the adaptive type 2 immune response during helminth infection and allergic inflammation via production of IL-13 and IL-5, as demonstrated in C57BL/6 mice^33,37,38^. IL-9 also contributed to the polarization of type 2 responses during infection of BALB/c mice with the protist parasite *L. major*^39^. As mast cells are also activated via the type 2 associated cytokine IL-3^31^ and Ab isotypes IgG1 and IgE^40^ during *Stronyloides* infection^4^, defective type 2 polarization could explain their reduced activation.

Indeed, we observed several significant changes in the cellular and humoral immune response to *S. ratti* infection in IL-9R-deficient mice. The antigen-specific type 2 cytokine production by MLN cells was reduced in day 6 *S. ratti*-infected C57BL/6 mice, either by trend (IL-3 and IL-13) or statistically significant (IL-4 and IL-9). A causative link between this decreased type 2 response and increased parasite burden appears likely at first glance. However, the fact that IL-9R^−/−^ BALB/c mice did not display any reduction in the antigen-specific production of IL-13, IL-4, IL-3 and IL-9 by MLN but still displayed elevated parasite burden in the intestine, argues against that. Furthermore, reduced type 2 cytokine production in IL-9R^−/−^ C57BL/6 mice was not correlated to reduced generation of type 2 associated Ab isotypes such as IgG1 or IgE. By contrast, IgG1 and IgE increased in the absence of IL-9R in C57BL/6 mice. Likewise, the reduced IgE induction in IL-9R^−/−^ BALB/c mice that could principally cause impaired expulsion of *S. ratti* from the intestine was not associated with reduction in other parameters of the type 2 immune response in these mice. Since serum concentrations of IgE were even elevated in IL-9R^−/−^ C57BL/6 mice, a causative link between changed IgE and elevated parasite burden that was observed in IL-9R^−/−^ mice of both genetic backgrounds, appears to be unlikely. Finally and despite the drastic suppression of intestinal IL-13 mRNA expression in IL-9R-deficient mice, the initiation of type 2 response to *S. ratti* infection as such was not defective as IL-4 and Arginase transcription increased during infection in WT and IL-9R^−/−^ mice on BALB/c and C57BL/6 background. Although Th17 cell expansion was shown to depend on IL-9 in C57BL/6 mice^41,42^, we observed no decrease but rather an increase in the cellular production of IL-17 in the absence of IL-9R in BALB/c and C57BL/6 mice. This contradiction may be explained by the finding that IL-9 also promotes Treg function in C57BL/6^43^ and BALB/c^44^ mice. Thus, depending on the context, deficiency of IL-9R or IL-9 may either lead to amelioration of Th17-mediated pathology such as EAE *in vivo*^41,42^ or induce aggravation of inflammation and disease, reflecting the impaired Treg function^43,44^.

In summary, the absence of IL-9R led to several changes in the adaptive immune response to *S. ratti* infection. The relevance of these changes, however, is difficult to interpret as they presented i) different on the different genetic background in BALB/c and C57BL/6 mice; ii) inconsistent regarding humoral and cellular responses; and iii) not correlated to infection outcome.

The gold standard of an efficient adaptive immune response is the establishment of protective immunity against a second infection. Although the resolution of *S. ratti* infection is not leading to sterile immunity, the number of intestinal parasites is more than 10-fold reduced in immune mice. The fact that protective immune memory was established to the same extent in IL-9R-deficient BALB/c and C57BL/6 mice as in WT mice, argues against a central and non-redundant contribution of IL-9 to the initiation of adaptive immunity to intestinal helminths such as *S. ratti*.

Our combined results rather suggest that the dominant function of IL-9 during intestinal *S. ratti* infection lies in promoting the activation of intestinal mucosal mast cells. Finally, taking into account a possible impact of the genetic background of the host, we further demonstrated that IL-9R deficiency delayed mucosal mast cell activation and elevated parasite burden in *S. ratti*-infected BALB/c and C57BL/6 mice to the same extent. This finding consents with our previous result that, again in BALB/c and C57BL/6 mice, mucosal mast cells are indispensable effector cells that mediate expulsion of *S. ratti* from the intestine but are not needed for initiation of adaptive memory responses^7^.

## Methods

### Ethics and mice

Animal experiments were conducted in agreement with the German animal protection law and experimental protocols were approved by Federal Health Authorities of the State of Hamburg. BALB/c and C57BL/6 IL-9Rα-deficient mice (termed IL-9R^−/−^ hereafter)^35^, a kind gift from Dr. Jean-Christophe Renauld, and wildtype BALB/c and C57BL/6 mice (termed WT hereafter) were bred in house and kept in individually ventilated cages under specific pathogen-free conditions. For all experiments, male and female mice were used at 7 to 10 weeks of age and experimental groups were matched for gender and age with 7 days variance.

### Parasitology

The *S. ratti* cycle was maintained in Wistar rats as described^30^ and infections were performed by s.c. injection of 2000 L3i in the hind footpad of mice. For analysis of protective memory, mice were vaccinated with 2000 irradiated L3i (160 Gy) 4 weeks before challenge infection with 2000 vital L3i. Quantification of parasite burden in the intestines of infected mice were performed as described^30^.

### mMCPT-1, humoral and cellular responses

For analysis of serum Ab and mMCPT-1, blood was collected from infected mice at the indicated time points and allowed to coagulate for 1 h at room temperature (RT). Serum was collected after centrifugation (10.000 × g) for 10 min at RT. *S. ratti*-specific serum Ig titres were quantified by ELISA, as described^30^. Polyclonal IgE concentrations were measured by using the IgE ELISA kit (BD, Heidelberg, Germany) and mMCPT-1 concentration was quantified using MCPT-1 Ready-SET-Go kit (eBioscience, San Diego, US) according to the manufacturer’s recommendations. For analysis of cellular responses, infected mice were sacrificed at day 6 p.i. and 1 × 10^6^ MLN cells were cultured in triplicate in 96-well round-bottom plates in RPMI 1640 medium supplemented with 5% FCS, 20 mM HEPES, L-glutamine (2 mM), and gentamicin (50 μg/mL) at 37 °C and 5% CO_2_, and stimulated for 72 h with medium alone, α-mouse CD3 (145-2C11, 1 μg/mL), or *S. ratti* L3 lysate (20 μg/mL). The cytokines in the culture supernatants were quantified by ELISA, as described^30^, using ELISA Kits from BioLegend. Detection limits with the standard dilution used in this study were <15 pg/mL for IL-9, IL-17, IL-4, IL-2 and IL-3 and <30 pg/mL and for IL-10, IL-13 and IFNγ.

### Bone marrow chimeras

Bone marrow was extracted from tibias and femurs of donor mice and 3 × 10^6^ bone marrow cells were i.v. injected into lethally irradiated (2 × 4 Gy doses, 3 hrs apart) recipient mice. Chimeras received 0.05% (v/v) Baytril (Bayer, Germany) in drinking water starting 1 week before until 4 weeks after transfer and were then left untreated for 8 weeks. Reconstitution was confirmed by flow cytometry.

### BMMC

BMMC were prepared as described^45^ and cultured in flasks 75 cm^2^ with 25 mL BMMC medium containing 10 ng/mL recombinant murine stem cell factor, 5 ng/mL recombinant murine IL-3 (both R&D Systems, Minneapolis, US) and for the first 2 weeks 10 µg/mL Ciprofloxacin (Bayer, Germany). 2 × 10^6^ cells per well were sensitized with dinitrophenyl (DNP)–specific IgE (clone SPE-7) 0.2 µg/mL (Sigma, Germany) or with naïve or immune mouse serum (1:100 dilution) in 12-well-plates over night at 37 °C. Cells were washed subsequently with PBS and 2 × 10^5^ cells per well were stimulated with PMA/Ionomycin (100 ng/mL), or DNP-HSA (20 ng/mL, both Sigma), or *S. ratti* L3 antigen lysate (100 µg/mL), or *S. ratti* adult antigen lysate (15 µg/mL) in round bottom 96-well-plates for 20 min at 37 °C. Immune serum for sensitization was a high titer serum derived from WT mice that were *S. ratti*-infected and re-infected after 30 days and sacrificed day 21 post second infection. Cells were stained with Alexa Fluor 647 labeled anti-mouse CD107a (LAMP1) (clone 1D4B), FITC labeled anti-mouse CD117 (clone 2B8) and PE labeled anti-mouse FcεRIα (clone MAR-1, all BioLegend, San Diego, US). Cells were analyzed by flow cytometry.

### RNA extraction, reverse transcription and qPCR

Total RNA was extracted from 1 cm of small intestine with TRIzol Reagent (Sigma-Aldrich, Deisenhofen, Germany) according to the manufacturer’s instruction. Two separate intestinal samples per mouse were pooled and analyzed. RNA concentration and purity was determined by Nanodrop 2000 c (Thermo Scientific, Wilmingto, USA). For qPCR analyses, cDNA was prepared from 1 µg of total RNA following DNaseI treatment using random primer and RevertAid H Minus Reverse Transcriptase (Thermo Scientific). 10 ng of cDNA were used for qPCR. Gene specific primers were as followed: **IL4** Fwd: TGA ACG AGG TCA CAG GAG AA, Rev: CGA GCT CAC TCT CTG TGG TG; **IL13** Fwd: AGA CCA GAC TCC CCT GTG CA, Rev: TGG GTC CTG TAG ATG GCA TTG; **Gapdh** Fwd: ATT GTC AGC AAT GCA TCC TG, Rev: ATG GAC TGT GGT CAT GAG CC; **Mcpt1** Fwd: TGC AGG CCC TAC TAT TCC TG, Rev: CCA TGT AAG GAC GGG AGT GT; **muc2** Fwd: ATG CCC ACC TCC TCA AAG AC, Rev: GTA GTT TCC GTT GGA ACA GTG AA; **Arg1** Fwd: CAG AAG AAT GGA AGA GTC AG, Rev: CAG ATA TGC AGG GAG TCA CC. qPCR was performed in duplicates in a Light Cycler 480 (Roche, Mannheim, Germany) or in a Corbett Rotor Gene 6000 Real-Time PCR Machine (QIAGEN, Hilden, Germany) using the following cycling conditions: 15 min polymerase activation at 95 °C, 45 cycles at 95 °C for 20 s, 60 °C for 15 s and 72 °C for 30 s. For each run a melting curve analysis was performed, to this end the temperature was ramped from 67 to 95 °C. Housekeeping gene GAPDH was used as internal control to calculate delta Ct. The relative expression of cytokine and protease genes during infection was determined with the comparative Ct method (ΔΔCt) using naïve mice as reference group.

### Statistical analysis

All data were assessed for normality and groups were compared by using Student’s *t*-test (parametric) or Mann-Whitney U test (non parametic) using GraphPad Prism software (San Diego, US). P values of ≤ 0.05 were considered to indicate statistical significance.

## Electronic supplementary material

Supplementary Material
