# Supplementary material for: Interleukin-9 promotes early mast cell-mediated expulsion of Strongyloides ratti but is dispensable for generation of protective memory
Source: Sci Rep. 2018 Jun 5;8:8636. doi: 10.1038/s41598-018-26907-2 (PMC5988711; doi:10.1038/s41598-018-26907-2)
Supplement: Supplementary file 1 — Supplementary Material [file 41598_2018_26907_MOESM1_ESM.pdf]

Supplementary Material

Interleukin-9 promotes early mast cell-mediated expulsion of *Strongyloides ratti* but is dispensable for generation of protective memory

Martina Reitz[1], Wiebke Hartmann[1], Nikolas Rüdiger[1,2], Zane Orinska[2], Marie-Luise Brunn[1] and Minka Breloer[1]

[1] Bernhard Nocht Institute for Tropical Medicine, Hamburg, Germany

[2] Division of Experimental Pneumology, Research Center Borstel, Borstel, Germany

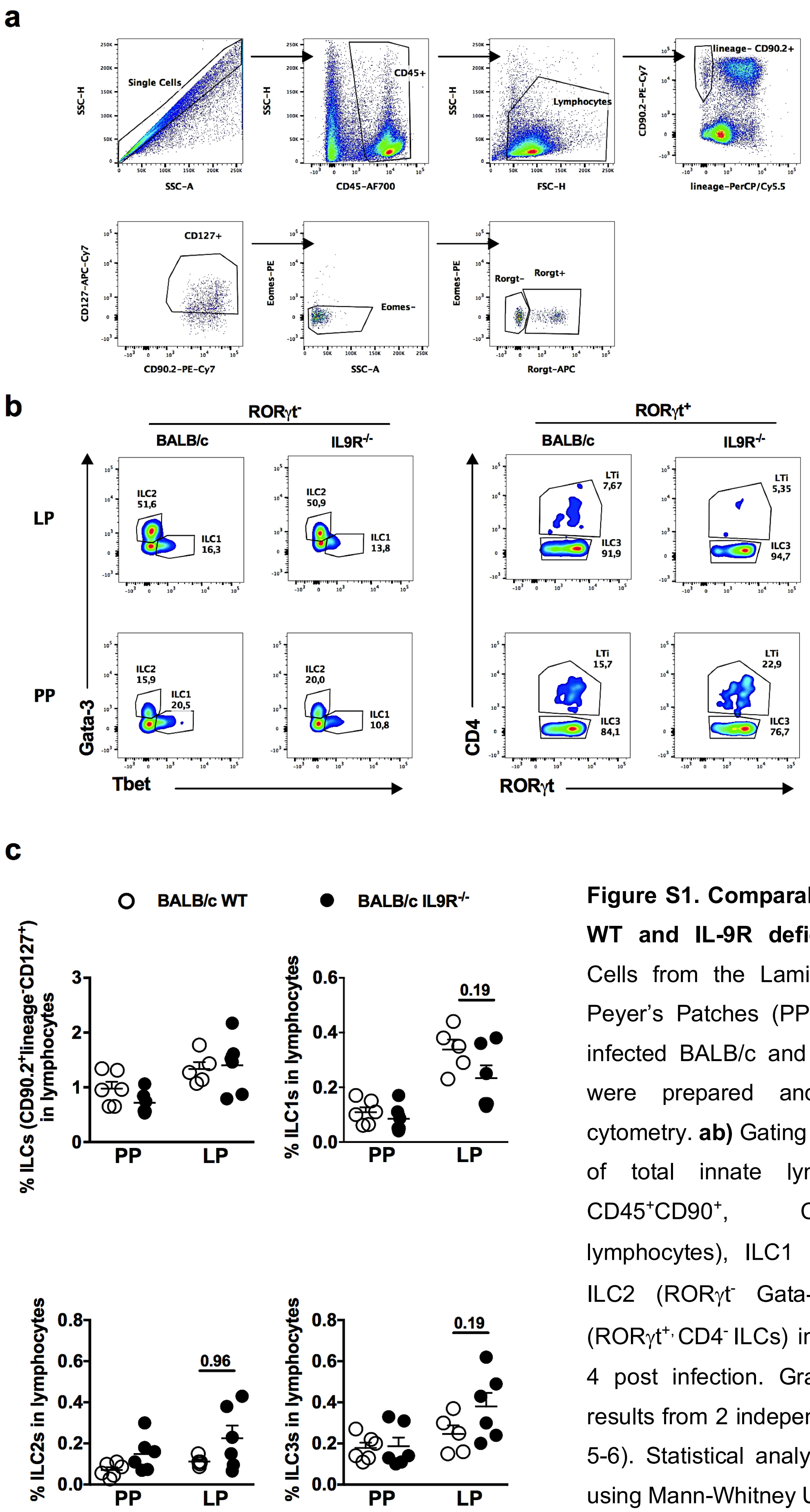

Figure S1. Comparable ILC expansion in WT and IL-9R deficient BALB/c mice. Cells from the Lamina Propria (LP) and Peyer's Patches (PP) from day 4 *S. ratti* infected BALB/c and BALB/c IL-9R$^{-/-}$ mice were prepared and stained for flow cytometry. **ab)** Gating strategy **c**) Frequency of total innate lymphoid cells (ILCs; CD45^{+}CD90^{+}, CD127^{+}, Eomes^{-} lymphocytes), ILC1 (RORγt$^{-,}$ Tbet^{+} ILCs), ILC2 (RORγt^{-} Gata-3^{+} ILCs) and ILC3 (RORγt$^{+,}$ CD4^{-} ILCs) in the LP and PP on day 4 post infection. Graphs show combined results from 2 independent experiments (n= 5-6). Statistical analysis was assessed by using Mann-Whitney U test.

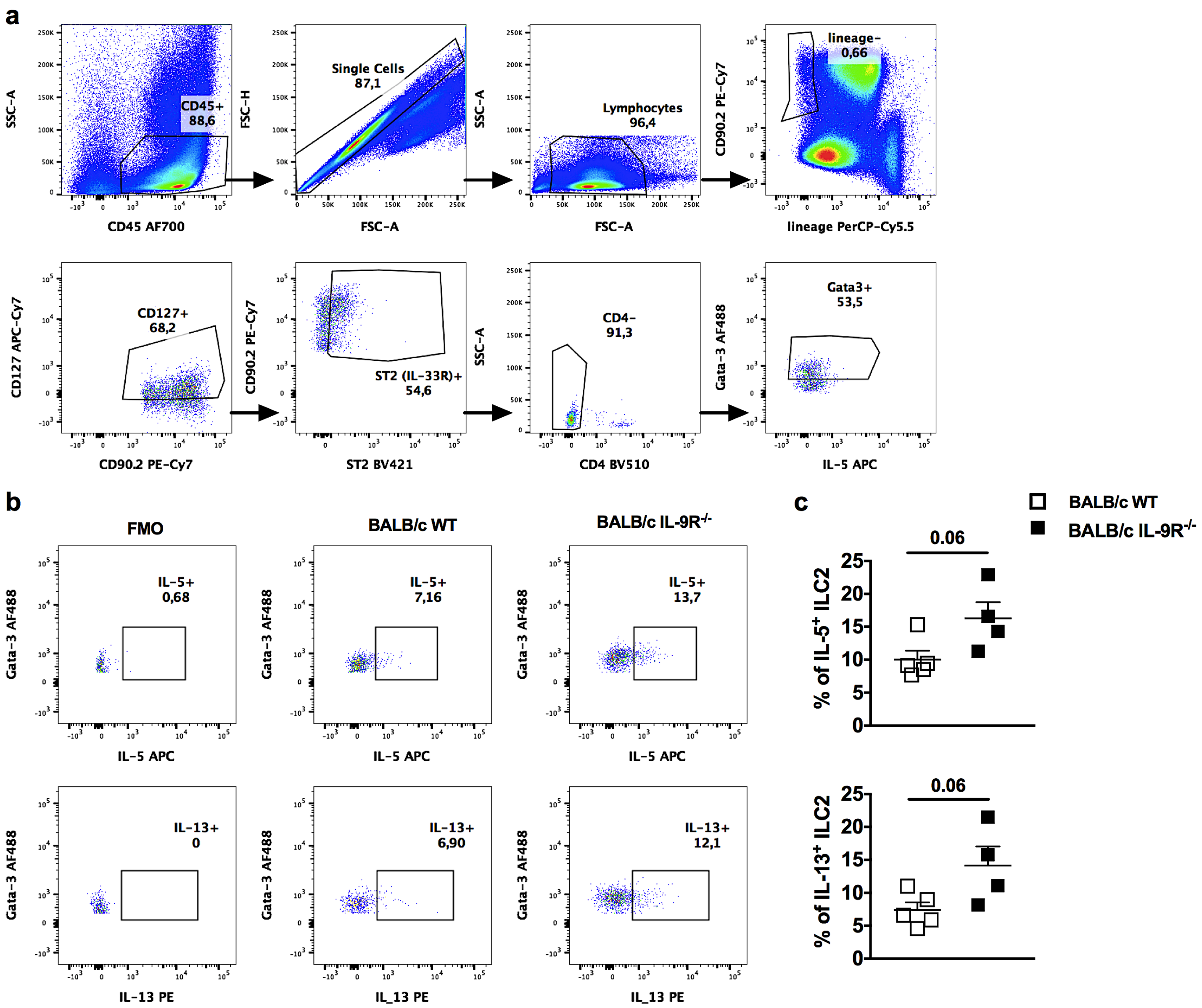

Figure S2. IL-5 and IL-13 production of lung ILC2 during *S. ratti* infection. Lung cells from day 6 *S. ratti* infected BALB/c and BALB/c IL-9R$^{-/-}$ mice were stained for flow cytometry after 4 h of PMA/Ionomycin stimulation at 37°C. Cells were stained for type 2 innate lymphoid cells (ILC2s) as CD45^{+}CD90^{+}, CD127$^{+,}$ ST2$^{+,}$ CD4^{-}, Gata-3^{+} lymphocytes, permeablized and stained for intracellular IL-5 and IL-13. **a)** Gating strategy. **b**) IL-5 and IL-13 positive ILC2; FMO: fluorescence minus one **c**) Percent of IL-5 and IL-13 positive ILC2s. Shown is one independent experiment (n= 4-6) and statistical analysis was performed by using Mann-Whitney U test.

Methods

Flow cytometry.

For surface staining, cells were stained for 30 min on ice in the dark with Biotin-labeled antibodies (lineage cocktail) against mouse CD11b (clone: M1/70), CD8 (clone: 53-6.7), CD19 (clone: 6D5), CD11c (clone: N418), CD3 (clone: 17A2), TCRβ (clone: B20.6), TCRγδ (clone: GL3), GR-1 (clone: RB6-8C5), CD5 (clone: 53-7.3), CD49b (clone: DX5), TER-199 (clone: TER-199) and NK1.1 (clone: PK136), BV421-labbeld anti-mouse ST2 antibody (clone: DIH9), APC-Cy7-labeld anti-mouse CD127 antibody (clone: A7R34) BV510-labeled anti-mouse CD4 antibody (clone: RM4-5), AF700-labeld anti-mouse CD45 antibody (clone: 30-F11) and PE Cy7-labeld CD90.2 antibody (clone: 30-H12). After washing, cells were stained with the secondary PerCP Cy5.5-labelled anti-Strep antibody for 30 min on ice in the dark. For intracellular staining cells were permeabilized with 250 µl fixation/permeabilization buffer for 30 min at 4°C, washed with permeabilization buffer and stained with the following antibodies: AF488-labeled anti-mouse Gata-3 (clone: L50-823; from BD), PE-labeled anti-mouse Eomes (clone: Dan11mag), PE/Dazzle594-labeld anti-mouse Tbet (clone: 4B10), APC-labeled anti-mouse RORγt (clone: Q31-378; from BD) APC-labeled IL-5 (clone: TRFK5) and PE-labeled IL-13 (clone: eBio13A) in permeabilization buffer. Ab were purchased from BioLegend or Affymetrix eBioscience if not otherwise stated. Samples were analyzed on a LSRII Flow Cytometer (Becton Dickinson) using FlowJo software (TreeStar).

Preparation of lung cells. Removed lungs were washed with PBS, cut into pieces and transferred into 2 mL RPMI 1640 with 10 % FCS , 1 % HEPES, 1 % Penicillin/Streptomycin. Liberase (final concentration 0.42 mg/mL) and DNase (final

concentration 100 µg/mL) were added and incubated for 45 min at 37°C in an incubator while rotating slowly in a roller. Suspension was filtered/mashed through a 70 µm nylon mesh. Cells were washed with washing buffer (1x PBS, 1% FCS, 1 mM EDTA), resuspended in 5m Percoll-37%-solution (RT) and centrifuged at 500 xg at RT for 20 min. Upper cell band and Percoll-solution were removed and erylysis was performed. After two washing steps with PBS cells were stained for flow cytometry.

Preparation of cells from Peyers's Patches and Lamina Propria. Intestine was isolated and Peyer's Patches were removed and kept in PBS. Intestine was transferred into a petri dish with washing buffer (1x PBS, 1% FCS, 1 mM EDTA) and cut open longitudinally to remove feces. After washing, the intestine was cut into 1 cm pieces and transferred into 20 mL washing buffer (37°C) and DTT was added to a final concentration of 1 mM. A magnetic stirring bar was added to the tube with the intestine and the tube was placed angular in beaker glass in a stirrer with medium rotation and incubated for 7 min at 37°C in an incubator. Suspension was then poured through a concave metal sieve and tissue was transferred into fresh washing buffer without DTT and incubated again on the stirrer for 7 min at 37°C. After pouring the suspension through a metal sieve tissue pieces were minced and transferred to 20 mL RPMI 1640 medium with 2 % FCS and digested with Collagenase VIII (final concentration 0.5 mg/mL) and DNase I (final concentration 20 µg/mL) for 30 min on a stirrer at 37°C. Cell solution was then mashed through a 70 µm nylon mesh and resuspended in 5 mL Percoll-37%-solution and centrifuged for 20 min at 500 xg, RT. Upper band and remaining Percoll was discarded and cells were washed with PBS and used for FACS staining. Peyer's Patches were mashed and cell solution was washed with PBS and stained for flow cytometry.

Cell stimulation with PMA/Ionomycin. Cells were stimulated in 1 mL RPMI 1640 with 10 % FCS, 1 % HEPES, 1 % Penicillin/Streptomycin and PMA/Ionomycin (final concentration 500 ng/mL, Merck) and Brefeldin A solution (final concentration 1x; Biolegend) for 4 h at 37°C in an incubator.
